# Mechanisms of white matter change induced by meditation training

**DOI:** 10.3389/fpsyg.2014.01220

**Published:** 2014-10-27

**Authors:** Michael I. Posner, Yi-Yuan Tang, Gary Lynch

**Affiliations:** ^1^Department of Psychology, University of OregonEugene, OR, USA; ^2^Department of Psychological Sciences, Texas Tech UniversityLubbock, TX, USA; ^3^Department of Anatomy and Neurobiology, School of Medicine, University of California, IrvineIrvine, CA, USA

**Keywords:** theta rhythm, myelination, diffusion tensor imaging, meditation, fractional anisotropy (FA)

## Abstract

Training can induce changes in specific brain networks and changes in brain state. In both cases it has been found that the efficiency of white matter as measured by diffusion tensor imaging is increased, often after only a few hours of training. In this paper we consider a plausible molecular mechanism for how state change produced by meditation might lead to white matter change. According to this hypothesis frontal theta induced by meditation produces a molecular cascade that increases myelin and improves connectivity.

In recent years there have been many reports of changes in white matter induced by training of human adults (see Zatorre et al., 2012 for a summary). Such changes usually involve training of specific networks involved in sensory discrimination, motor activity, or working memory. We call this form of training network training because it uses a specific task to induce changes in the underlying brain network. A different form of training involves training the brain state as occurs in the use of aerobic exercise or meditation, which we call state training (Tang and Posner, 2009; Tang et al., 2012b).

Our studies used a form of mindfulness meditation, integrative body-mind training (IBMT) in comparison with relaxation training (RT), which served as an active control (Tang et al., 2007). We used diffusion tensor imaging (DTI) before and after 4 weeks of training with IBMT and RT (Tang et al., 2010). We found significantly greater increases in fractional anisotropy (FA) following IBMT than after the RT control. The training effect was in white matter pathways connecting the anterior cingulate cortex (ACC) to other brain areas (Tang et al., 2010). We also found that after 2 weeks the FA change was entirely due to axial diffusivity (AD), which declined significantly more following IBMT than RT (Tang et al., 2012a). AD is thought to relate to changes in axonal density (Kumar et al., 2010, 2012). After 4 weeks FA involved changes in both axial and radial diffusivity (RD). RD is thought to reflect myelination (Song et al., 2002, 2003). This evidence suggests that meditation can influence brain areas known to be involved in self control in children and adults (Posner and Rothbart, 2007).

How can white matter change in as little as 2–4 weeks of training in meditation? In this paper we use evidence from a variety of human and animals studies to examine how meditation influences frontal brain rhythms, and the consequence of these rhythms on protease secretion that influence glial cells in forming the basis of white matter change. These mechanisms are summarized in **Figure 1** and elaborated below. We believe that they represent one way changes in brain state induced by meditation could lead to the observed changes in white matter as shown by DTI.

**FIGURE 1:**
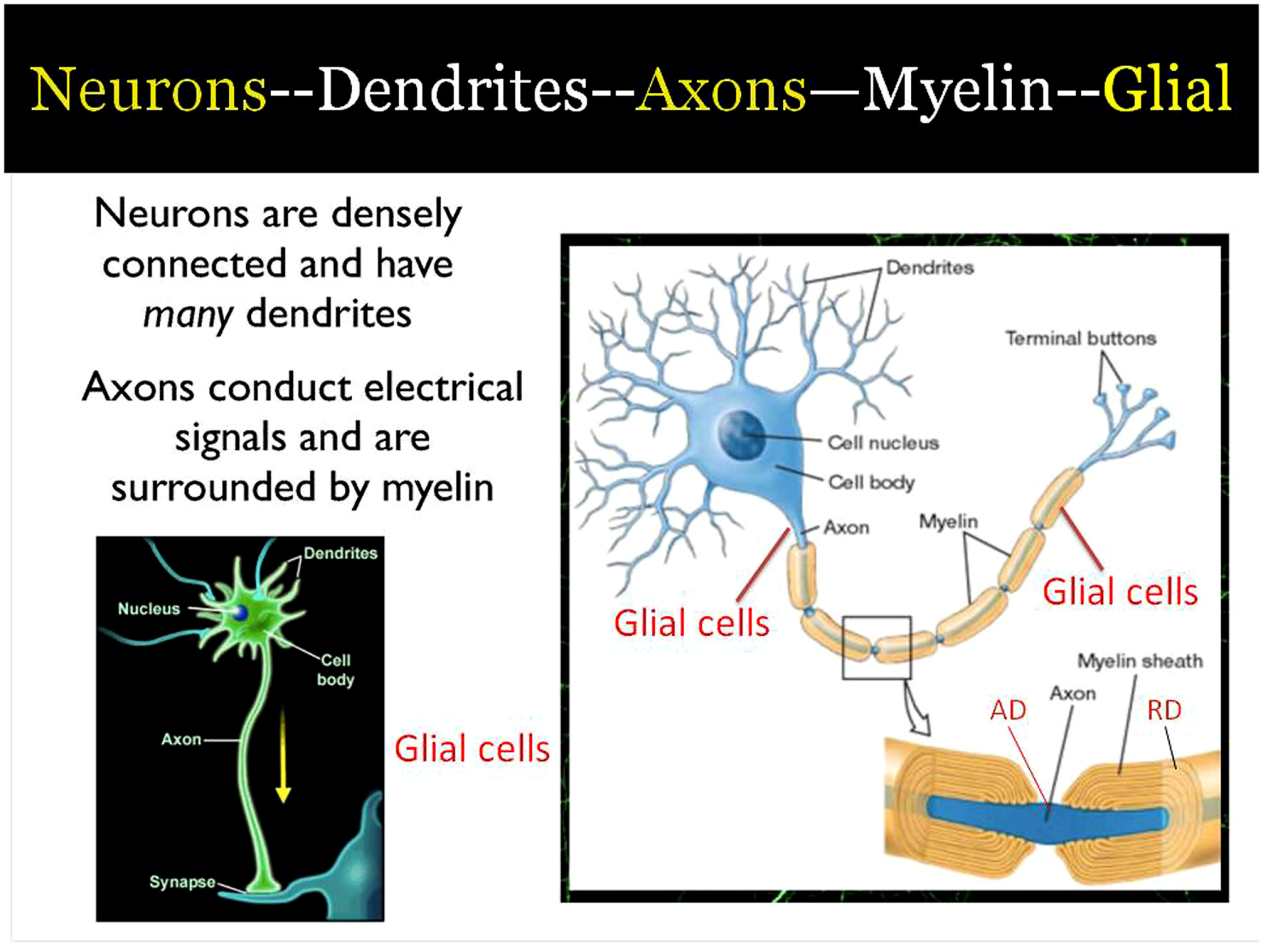
**Summary cartoon of major areas of change in the formation of white matter due to training.** (AD, axial diffusivity; RD, radial diffusivity).

## INCREASED FRONTAL THETA

Frontal theta has been regarded as a mechanism for recognizing the need for cognitive control and providing information to other brain areas (Cavanagh and Frank, 2014). The meditative state produces an increase in the EEG theta rhythm over frontal electrodes consistent with a generator in the anterior cingulate (Cahn and Polich, 2006; Chiesa and Serretti, 2010). Increased frontal midline theta is thought to reflect positive emotional state, internalized attention and autonomic nervous activity. Using IBMT with 1 week of training we recently replicated this result in frontal midline electrodes consistent with ACC activation (Tang et al., 2009; Xue et al., 2014). Theta oscillations may also induce changes in posterior alpha rhythms in humans (Song et al., 2014).

Theta rhythm in the ACC is also triggered by the violation of expectation such as when a person is presented with a novel event (Berger, 2012, p. 30). It has also been found that presenting a novel event within a visual search paradigm tends to recruit the ACC (Shulman et al., 2009). We have speculated that the presentation of a novel visual event may serve to foster the connectivity of the ACC, which has been found to increase during development of infants and young children (Posner et al., 2012).

Bursts of transcranial magnetic stimulation (TMS) in the theta range have been shown to induce changes in plasticity in the human motor cortex that outlast the stimulation (Huang et al., 2006). Recently theta burst rTMS was shown to increase cortical excitability and resting state connectivity in the motor system in a dose dependent manner (Nettekoven et al., 2014). Similar theta burst TMS has been shown to influence cognitive control networks including the ACC (Gratton et al., 2013). In a rat model repeated theta burst stimulation induced changes in calbindin protein in the frontal lobe thus reducing inhibitory control of neuronal spiking (Volz et al., 2013). These findings may provide the opportunity to test the role of theta in animal models.

## INDUCTION OF PROTEASE CALPAIN

How can theta activity in mid frontal brain areas be related to changes in white matter connecting the ACC to other brain areas? Bouts of high frequency neuronal firing synchronized to theta are known to activate the calcium sensitive protease calpain, an event that causes substantial changes to the neuronal sub-membrane cytoskeleton (Vanderklish et al., 1995, 2000). Because they are structural in nature, the modifications produced by the protease can be very long lasting. These effects are usually discussed in terms of synapses and the extraordinarily persistent long-term potentiation effect (Lynch, 1998). This role for calpain in learning and memory through inducing synaptic plasticity has been disputed, but recent studies seem to indicate that it plays an important role in learning and memory (Zadran et al., 2010). Studies of drugs reducing calpain in mice (Amini et al., 2013) show impairment of dendritic spines and of long term potentiation.

The evidence for the role of calpain in learning and memory supports the observation that it can induce axonal growth and thus foster brain connectivity. Notably, calpain is also found in axons where it appears to play an important role in growth (Qin et al., 2010). It has been known for some time that action potentials trigger an influx of calcium into axons (e.g., Zhang et al., 2006), with recent work demonstrating activity-driven stimulation of calpain (Huff et al., 2011). The latter event, which modifies the same primary membrane cytoskeletal protein affected by theta bursts at synapses (Vanderklish et al., 2000), is followed by a substantial change to the myelin sheath (Huff et al., 2011).

Moderate stimulation of the protease during repeated, prolonged episodes of theta could, therefore, (i) foster brain connectivity and (ii) alter the axon-glia relationship; together, these lasting effects constitute plausible contributors to the results obtained with DTI. Of interest, recent work emphasizes the possibility that changes in oligodendrocytes contribute importantly to memory and cognition (Fields, 2013).

## RAPID WHITE MATTER CHANGES

Traditionally white matter has been thought not to change after the period in development when axonal migration and myelination have taken place. However, this concept has been changing. For example, adult mice show reduced myelin thickness in prefrontal cortex induced by social isolation (Liu et al., 2012). The loss of myelin in the isolated mice, was associated with the presence of oligodendrocytes with immature nuclear chromatin. Beirowski (2013) argues that in adults axons are enwrapped by glia, including oligodendrocytes, with which they closely interact to form a unique symbiotic unit, a key contributor to the normal function of axonal connections. Indeed parts of the glia may respond to axonal damage within a few minutes (Guertin et al., 2005). Two key components of this final pathway could be axonal transport failure and calcium influx that among other targets activate cysteine proteases such as calpains whose inhibition also confers protection of injured axons *in vitro* and *in vivo* (Ma, 2013; Ma et al., 2013). While calpains are often associated with rapid release after axonal injury, they have also been associated with axonic growth within the axon rich intermediate zone of the cortex (Spira et al., 2003; Yang et al., 2011). Moreover, blocking of these molecular events interferes with this growth (Yang et al., 2011). Spira et al. (2003, p. 311) suggest “in light of the present findings, it would be interesting to examine the role of calpain ... in events that lead to morphological restructuring of neurons in relation to plasticity such as in learning and in memory acquisition processes.” To our knowledge these critical studies have not yet been done, but as described above, calpain provides a viable possibility for white matter changes in brain state training.

Changes in axonal growth or myelination via calpain are not the only molecular route to alteration of myelin. For example, in one study mutant mice without erbB signaling from Neuregulin 1 showed demyelination and thinner myelin sheaths as adults (Chen et al., 2006). As expected these mice showed slower conduction velocities that would result in slower reaction times. However, these effects depend upon a mutation and would not result from learning. In any case, our goal is more to describe one possible route between training effects and white matter changes and we think other mechanisms are likely. Other potential mechanisms of white matter change are discussed in a recent review (Zatorre et al., 2012), but they are not clearly related to meditation effects.

## TRAINING INDUCED WHITE MATTER CHANGES

Magnetic resonance imaging (MRI) cannot examine molecular events. However, the directionality of water molecules along white matter pathways (FA) can be measured and is believed to reflect the efficiency of connections. Changes in FA found in many learning studies can be linked to a plausible set of events including increased frontal theta and activation of the protease calpain which in turns works through glia cells to change myelin. Moreover the ability to impose the theta rhythm by brain stimulation may allow testing of its effects without extensive training.

A number of recent learning studies have shown FA changes that may take advantage of the molecular cascade that we have been describing. Most of these studies have involved learning of specific networks, for example, working memory training (Takeuchi et al., 2010), or juggling (Scholz et al., 2009). The periods of training run from a few hours to many months. In most studies of network training these effects are said to lead to an increase in myelination and changes in RD. However, our studies of meditation have shown that within 2 weeks of training there are changes in AD, most often associated with axonal density, and after 4 weeks both AD and RD are changed, suggesting both axonal density and myelin changes. We do not know if the differences between studies relate to state training vs. network training or other differences. It is possible that the study of calpain and other possible pathways for inducing these rapid changes will provide a better understanding of the time course of various forms of increased efficiency in the connectivity between brain areas.

## Conflict of Interest Statement

The authors declare that the research was conducted in the absence of any commercial or financial relationships that could be construed as a potential conflict of interest.
